# Prevalence of diabetes mellitus and impaired glucose tolerance in a rural community of Angola

**DOI:** 10.1186/1758-5996-2-63

**Published:** 2010-11-01

**Authors:** Antonio D Evaristo-Neto, Maria Cristina Foss-Freitas, Milton C Foss

**Affiliations:** 1 Department of Internal Medicine, Endocrinology and Metabolism Division, Ribeirào Preto School of Medicine, Sào Paulo University, Brazil

## Abstract

**Background:**

To determine the prevalence of diabetes mellitus (DM) and impaired glucose tolerance (IGT) in a rural community (Bengo) of Angola.

**Methods:**

A random sample of 421 subjects aged 30 to 69 years (30% men and 70% women) was selected from three villages of Bengo province. This cross-sectional home survey was conducted using a sampling design of stage conglomerates. First, clinical and anthropometric data were obtained and fasting capillary glucose level was determined. Subjects who screened positive (fasting capillary glucose ≥ 100 mg/dl and < 200 mg/dl) and each sixth consecutive subject who screened negative (fasting capillary glucose < 100 mg/dl) were submitted to the second phase of survey, consisting of the 75-g oral glucose tolerance test. Data was analyzed by the use of SAS statistical software.

**Results:**

The prevalence rates of diabetes mellitus and IGT were 2.8% and 8.1%, respectively. The age group with the highest prevalence of diabetes was 60 to 69 years (42%). Impaired glucose tolerance prevalence was 38% in the 40 to 49 year age group and it increased with age, considering that the 50 to 59 and 60 to 69 year age groups as a whole represent 50% of all subjects with impaired glucose tolerance. The prevalence of diabetes mellitus did not differ significantly between men (3.2%) and women (2.7%) (p = 0.47). On the other hand, the prevalence of impaired glucose tolerance among women showed almost twice that found in men (9.1% vs. 5.6%, respectively). Overweight was present in 66.7% of the individuals with diabetes mellitus and 26.5% of individuals with impaired glucose tolerance showed overweight or obesity.

**Conclusions:**

Although the prevalence of diabetes mellitus was low, the prevalence of impaired glucose tolerance is considered to be within an intermediary range, suggesting a future increase in the frequency of diabetes in this population.

## Background

Over the past century, diabetes mellitus was considered to be a rare medical condition in Africa. However, epidemiological studies carried out in the 90's have provided evidence of a different picture [1,2]. The prevalence of type 2 diabetes is high among Africans Americans, Afro-caribbeans and among African migrants in Europe, all of them sharing genetic ancestry with black Africans [3-5]. Diabetes and its long-term complications are higher in populations of African origin who have migrated to Western countries compared to Caucasians living in the same countries. Also, because of the adoption of Western lifestyles there is a global trend towards an increased incidence and prevalence of diabetes mellitus in Africa [2,6,7]. Indeed, Africa is experiencing one of the most rapid demographic and epidemiological transitions of the world history, characterized by a rise in the burden of non-communicable diseases. A prospective approach to the burden and prevention strategies has been hindered by the scarcity of data on diabetes in Africa [2,8]. The number of people with diabetes is increasing due to population growth, aging, urbanization, and increasing prevalence of obesity and physical inactivity [9]. Epidemiological studies of non-communicable diseases are rare in Africa and there is a need for more information on the prevalence of these diseases in this region of the world [2]. Quantifying the prevalence of diabetes and the number of people affected with diabetes is important for rational planning and adequate allocation of resources [9,10].

The main objective of this population-based study was to determine the prevalence of diabetes mellitus and impaired glucose tolerance in a rural community (Bengo) of Angola. It was also analyzed the associations of glucose metabolism disturbances with body adiposity and/or hypertension.

## Methods

A cross-sectional study was conducted in Caxito municipality, a training community of the Public Health Department of Agostinho Neto University School of Medicine. Caxito, the capital of Bengo province, is located 60 km north of Luanda, the capital of Angola. Its estimated population is of 54,592 with 54% of women and a predominant Bantu ethnic group. Caxito is divided in 15 villages with rural characteristics. The study was approved by the Scientific Council of Agostinho Neto University School of Medicine. Four medical students were trained as interviewers. Administrative authorities of Caxito municipality were informed and approved the study. Village populations were informed and invited to participate through meetings and advertisements via local radio broadcasting.

The minimum sample size of 400 people was calculated by estimating a possible prevalence of diabetes of 0.7%, with an acceptable 95% CI and 1% maximum error. Sampling was undertaken using the conglomerate stage [11]. We randomly selected three rural villages. A two-stage survey was carried out. First, men and non-pregnant women aged 30 to 69 years were invited to participate in the study. After informed written consent was obtained, selected participants were asked to fast overnight. On the next day, a standard questionnaire was applied for identification data, and to collect anthropometric and clinical data. The subjects were then submitted to medical examination including the measurement of arterial blood pressure (BP) and anthropometric (weight, height, BMI = W(kg)/H^2^(m)) data. Capillary blood from a finger puncture was immediately analyzed for fasting blood glucose concentration by glucose oxidase method using an portable electronic blood glucose monitor (Accu Chek Advantage^®^) with commercially available strips (Dextrotix^®^).

Individuals with fasting capillary glycemia (FCG) ≥ 100 mg/dl and < 200 mg/dl (positive screening test) were immediately submitted to a 75 g oral glucose tolerance test and capillary glucose was measured 2 hours later (second phase of the study). Every sixth consecutive individual who screened negative (FCG < 100 mg/dl) was also submitted to the glucose load test. Previously-diagnosed individuals and those with fasting or two-hour capillary glycemia ≥ 200 mg/dl were considered to have diabetes mellitus. Individuals with two-hour capillary glycemia ≥ 140 mg/dl and < 200 mg/dl were considered to have impaired glucose tolerance (IGT) and those with FCG < 140 mg/dl were considered to have normal glucose tolerance. Individuals with systolic BP (SBP) ≥ 140 mmHg and diastolic BP (DBP) ≥ 90 mmHg or taking an antihypertensive medication were considered to be hypertensive according to WHO/ASH(2003). BMI between 18.5 to 24.9 was considered normal, 25 to 29.9 overweight and equal or higher than 30 obese.

Data was analyzed using the SAS statistical software. The difference between men and women was calculated by the Fischer test. The results are shown as mean and standard deviation or as percentage. The level of significance was set at 5%.

## Results

A total of 1716 people living in 245 selected residential address were registered, but 1282 were not eligible for the study: 1205 of them (70.2%) because they were aged < 30 years, 21 (1.2%) because they were aged ≥ 70 years and 56 (3.3%) due to pregnancy. Initially, 434 subjects were enrolled but 13 subjects (3.0%) were not at home on the day of the interview, the only reason of lack of participation in the study. The response rate was 97%, which indicates the active cooperation of the eligible participants. Thus, 421 subjects participated in the first phase of the study and 130(30.8%) in the second phase, 46 (35.3%) of them as control (corresponding to the each sixty consecutive subject who screened negative).

**Figure 1:**
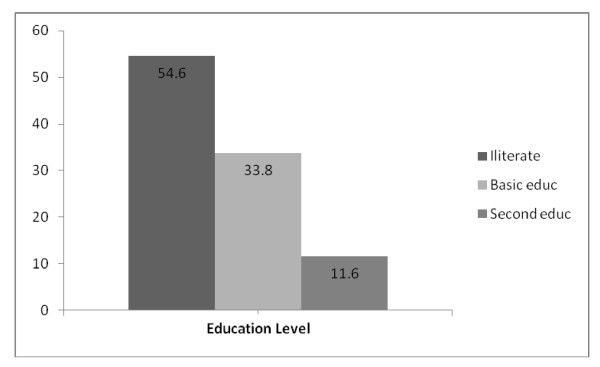
**Educational status of individuals in the study sample**.

According to education level, 230 individuals (54.6%) were illiterate, 142 (33.8%) had basic level education and 49(11.6%) had secondary level education (Figure 1). No one had university education. With exception of only one individual classified as mixed skin color, 99.8% of individuals were of black skin color. Previously diagnosed diabetes mellitus was only known by one subject (8.0% of all affected subjects with diabetes) on oral anti-diabetic drug therapy. The majority of the subjects studied (70%) were females and gender age group distributions were quite similar, ranging between 22% and 28%.

Of the 421 subjects tested (30% men and 70% women), 375 (89.1%) were classified as individuals with normal glucose tolerance, 34 (8.1%) as having impaired glucose tolerance, and 12 (2.8%) were diagnosed with diabetes mellitus. Impaired fasting glycemia was verified in 51 individuals(12.1%). The overall prevalence of diabetes mellitus in the study sample was 2.8% (95% CI 1.2-4.4) and the overall prevalence of impaired glucose tolerance was 8.1% (95% CI 5.5-10.7) (Table 1). Among 12 individuals who met the criteria for diabetes mellitus, 66.7% were females and 33.3% were males. Diabetes was diagnosed by the determination of fasting blood glucose in seven subjects (58%) and by the 75 g oral glucose tolerance test in the remaining five subjects (42%).

**Table 1:** Prevalence of diabetes mellitus and impaired glucose tolerance in the study sample

Categories	Total			Women			Men		
	N	%	CI(95%)	N	%	CI(95%)	N	%	CI(95%)
DM	12	2.8	1.2-4.4	8	2.7	1.2-4.2	4	3.2	1.4-4.9
IGT	34	8.1	5.5-10.7	27	9.1	5.2-11.3	7	5.6	3.6-6.7
No DM-No IGT	375	89.1	86.1-92.2	262	88.2	85.2-91.6	113	91.2	88.2-94.6

The prevalence of diabetes mellitus did not differ significantly between men (3.2%,95%CI 1.4-4.9) and women (2.7%,95%CI 1.2-4.2) (p = 0.47). On the other hand, the prevalence of impaired glucose tolerance among women showed almost twice that found in men (9.1% vs. 5.6%, respectively, p = 0.30). Of the 130 individuals submitted to the OGTT, five (4%) screened positive for diabetes mellitus, 34 (26%) screened positive for impaired glucose tolerance and 91 (70%) were classified as having normal glucose tolerance. Of a total of 91 individuals whose 75 glucose tolerance tests were normals, 38 (41.8%) showed previous fasting glycemia < 100 mg/dl, 47(51.6%) showed previous fasting glycemia ≥ 100 and < 126 mg/dl, and 6 (6.6%) showed previous fasting glycemia ≥ 126 and < 200 mg/dl.

The mean age of individuals with diabetes mellitus was 54.3 ± 10.7 years (males: 53.5 years vs. females: 54.6 years) and the mean age of the study sample as a whole was 49.6 ± 11.3 years (males: 48.7 years vs. females: 49.9 years). Most of the individuals with diabetes mellitus were 50 to 69 years (58%), whereas most of those without diabetes mellitus or impaired glucose tolerance were 30 to 49 years old (52%). No difference was found in the age group distribution of individuals with impaired glucose tolerance. All the individuals diagnosed as having diabetes mellitus had an unknown family history.

**Figure 2:**
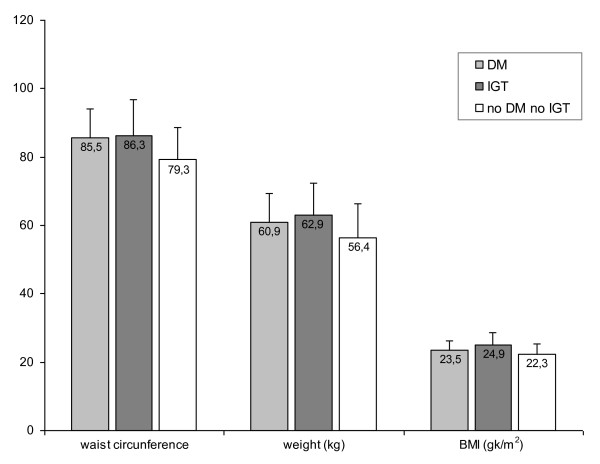
**Anthropometric data (waist circumference, weight and BMI) of individuals with diabetes mellitus (DM) or impaired glucose tolerance (IGT) and without DM or IGT (noDM noIGT)**.

Although the mean values of anthropometric data verified in the study sample were within the normal range, subjects with impaired glucose tolerance or diabetes mellitus had slightly higher values than those observed in the subjects without diabetes mellitus or impaired glucose tolerance (Figure 2). Overweight was present in 66.7% of the individuals with diabetes mellitus and 26.5% of individuals with impaired glucose tolerance showed overweight or obesity. Overweight or obesity was present in only 15.4% of the whole sample. Agricultural work was the most frequent occupation (76.7% of participants).

Hypertension was found in 38.7% of the study sample as a whole (37.6% among the subjects without DM or IGT) and in 47.8% of individuals with diabetes mellitus or impaired glucose tolerance.

## Discussion

The prevalence of diabetes mellitus and impaired glucose tolerance in the province of Bengo was 2.8% and 8.1%, respectively, which is similar to that found in sub-Saharan countries of Africa except South Africa, where higher prevalence were found. This was the first study of diabetes mellitus carried out in Angola, and therefore there are no previous data for comparison. The prevalence of diabetes mellitus found in this rural community of Angola allowed us to estimate a higher number of individuals with diabetes compared to that estimated by WHO, based on extrapolation of data from Central Africa countries like Cameroon whose rural prevalence of diabetes mellitus determined according to the same diagnostic criteria was 0.7% in 1996 [1].

As in other African rural communities, the prevalence of diabetes mellitus found in the present study was much lower than that found in African migrant communities, African Americans, Caribbean Africans in United Kingdom and Caucasians [3-5]. Complex carbohydrate consumption and high energy expenditure by agricultural workers lead not only to a low obesity prevalence but also to a low prevalence of diabetes. In addition, the low life expectancy in sub-Saharan African countries, except South Africa, contribute for this scenario [10,12,13]. In fact, Angola which life expectancy at birth of 39 years (men) and 41 years (women) [14,15], has a low number of people aged ≥ 40 years, a situation reflected on age group distribution of individuals with diabetes mellitus and impaired glucose tolerance in the present study. The decreasing number of elderly people as a result of high mortality of people with diabetes mellitus due to lack of health care and to the HIV/AIDS epidemic may also contribute to this low prevalence of diabetes mellitus[14,15].

Ninety-two percent of all cases of diabetes found were unknown before the survey. This rate is much higher than those reported in similar studies in other developing countries (the global average is around 50%) [16] and may reflect less awareness to the disease and a lack of healthcare. Thus, there is a need for screening prevention programs on scientific and political agenda.

Some factors such as stress related to the long civil war, body loading with salt from consumption of products using a salt conservative method and psychological aspects could explain the high prevalence of hypertension in this community (38.7%). Hypertension has been found to be high in African populations with and without diabetes[7,17].

According to King and Rewers [18], the prevalence of diabetes mellitus in the rural community studied is low (< 3%) but the prevalence of impaired glucose tolerance is within the intermediate range (3-10%), a fact that may imply a trend toward an increasing prevalence of diabetes in the future. The prevalence of overweight and obesity was also low. Hypertension appeared to be an important associated pathological situation not only in individuals with diabetes mellitus but also in those with impaired glucose tolerance.

## Conclusions

Although the prevalence of diabetes mellitus was low, the prevalence of impaired glucose tolerance is considered to be within an intermediary range, suggesting a future increase in the frequency of diabetes in this population.

## Competing interests

The authors declare that they have no competing interests.

## Authors' contributions

ADEN, MCFF and MCF participated in the design of the study. ADEN and MCFF performed the data collection and the statistical analysis. ADEN, MCFF and MCF wrote the paper. All authors read and approved the final manuscript.
